# Impact of lead exposure on the thyroid glands of individuals living in high- or low-lead exposure areas

**DOI:** 10.1097/MD.0000000000033292

**Published:** 2023-03-24

**Authors:** José Estefano Rivera-Buse, Sheila Jissela Patajalo-Villalta, Eduardo Antônio Donadi, Fernando Barbosa, Patrícia Künzle Ribeiro Magalhães, Léa Maria Zanini Maciel

**Affiliations:** a Medicine School, Medical Sciences Faculty – Universidad Central del Ecuador, Quito, Ecuador; b Department of Internal Medicine, Ribeirão Preto Medical School – University of São Paulo, Ribeirão Preto, São Paulo, Brazil; c Medicine Faculty – Pontifical Catholic University of Ecuador (PUCE), Quito, Ecuador; d Department of Internal Medicine, Hospital Vozandes, Quito, Ecuador; e Department of Clinical, Toxicological, and Bromatological Analysis, Ribeirão Preto Pharmaceutical Sciences School, Ribeirão Preto, University of São Paulo, São Paulo, Brazil.

**Keywords:** free-thyroxine, lead, thyroid cancer, thyroid nodule, thyroid-stimulating hormone

## Abstract

Ecuador was an endemic area for iodine deficiency; however, due to the population consumption of iodized table salt, the country is nowadays considered iodine sufficient. Despite the population consumption of iodized salt for more than 50 years, the prevalence of hypothyroidism has increased in recent years. A similar increment has been reported for thyroid cancer (TC) becoming the second most common cancer in women and seventh most common cancer in men. High blood lead (BPb) level is a controversial causal factor for impaired thyroid function as well as a debated environmental cause for the increased incidence of TC. To study the association between BPb and thyroid function, anti-thyroid peroxidase (anti-TPO) and anti-thyroglobulin (anti-Tg) antibodies, and the presence of benign and malignant thyroid nodules in Ecuadorian individuals living in high lead exposure (HE) areas compared with those living in low lead exposure (LE) area. We evaluated 197 euthyroid individuals: 70 from Esmeraldas (close to a petrol refinery) and 27 from La Victoria de Pujilí (Pb-glazing ceramics), considered HE areas, and 100 from Quito, considered the LE area. In parallel, we evaluated 187 patients with hypothyroidism (60, 27, and 100 patients from Esmeraldas, Pujilí, and Quito, respectively). BPb was detected using atomic absorption spectroscopy, while thyroid-stimulating hormone (TSH), free-thyroxine (FT4), and autoantibodies were measured using chemiluminescence assays. Thyroid ultrasonography was performed in 300 individuals and fine-needle aspiration biopsy (FNA) was performed only when required based on the guidelines of the American Thyroid Association. The BPb levels (mean ± SD) in the HE areas were increased (8.5 ± 7.4) than those in the LE area (3.2 ± 2.4*, P* < .001). No significant associations were observed between BPb and TSH, FT4, or thyroid antibody levels. Enlarged thyroid glands and larger thyroid nodules were primarily observed in HE areas. Just 1 TC was observed. High BPb levels detected in HE areas were not associated with thyroid function or thyroid autoantibodies; however, increased thyroid size and numbers of thyroid nodules were observed, demanding further actions to control lead contamination in these Ecuadorian areas.

## 1. Introduction

Because of the population consumption of iodized table salt, Ecuador is nowadays considered an iodine sufficient country.^[1]^ Despite the population consumption of iodized salt for more than 50 years, the prevalence of hypothyroidism has increased in Ecuador.^[2,3]^ A similar increment has been reported for thyroid cancer (TC). According to the Ecuadorian Society to Combat Cancer, in 2017, TC was the second most frequent cancer in women (41 per 100.000 women) after breast cancer, while TC was ranked seventh in men (7.9 per 100.000).^[4]^

In Ecuador, there are populations living in areas highly exposed (HE) to lead (Pb), one of which is the city of Esmeraldas, where the Esmeraldas State Refinery is located. According to a report by the General Comptroller of the State, lead tetraethyl is no longer added to gasoline to improve its performance; however, this product has remained in storage for more than 16 years at Esmeraldas State Refinery, constituting an additional permanent pollutant in Esmeraldas.^[5]^ Another Ecuadorian HE population is the parish La Victoria de Pujilí, exhibiting historical exposure to inorganic lead, due to the use of Pb-glazing ceramic cookware industry.^[6]^ The Centers for Disease Control and Prevention has established that the blood lead (BPb) level in adults should be <10 μg/dL.^[7]^

Some studies reported that lead can cause functional deterioration of the pituitary-thyroid axis, modifying the thyroid physiology,^[8]^ and the chronic lead exposure can cause anatomic-histological changes in thyroid gland, such as decrease in the size of thyroid follicles and alteration of the follicle cell nucleus.^[9]^ In recent years, several studies indicate that lead affects the thyroid function,^[10]^ causes a decrease in the production of tetraiodothyronine (T4) and elevation of thyroid-stimulating hormone (TSH).^[11]^ Other authors reported a decrease of T4 without affecting triiodothyronine or TSH,^[9,10]^ apparently, due to the inhibition of the 5 deiodinase type 1 enzyme. These controversial results have been related to different BPb levels and to the mode of lead exposure.^[9–11]^ Additionally, some authors have indicated that there is an inverse relationship between BPb and TSH in hypothyroid women, but not in men^[12]^; however, other studies have not reported such a relationship.^[13]^ It has also been proposed that lead can increase the risk of developing TC.^[14]^ Notably, most studies associating environmental pollutants and thyroid function were performed in men who are professionally exposed to these pollutants.^[15]^ Therefore, the effect of lead on the thyroid gland remains controversial.

Considering the high incidence rate of TC in Ecuadorian women, in this study, we evaluated the following: the BPb levels, the relationship between BPb and thyroid function, and thyroid autoantibody levels, and the frequency of benign and malignant thyroid nodules in euthyroid and hypothyroid individuals living in Ecuadorian areas exhibiting different lead exposure, that is, high (HE) or low exposure (LE) areas, primarily encompassing women.

## 2. Methods

### 2.1. Study population

From 2019 to 2021, we studied 197 (181 women) adult healthy euthyroid individuals (mean ± SD = 49.6 ± 14.6 years) and 187 (171 women) hypothyroid patients (51.6 ± 15.3 years) from 2 Ecuadorian areas known to be highly exposed to lead, including individuals living <5 kilometer from the Esmeraldas petrol refinery, which has been installed in the city for more than 50 years, and the parish of La Victoria del Pujilí, where lead, tin, and copper contaminations come from the continuous practice of glazing tiles, handicrafts, and kitchen utensils, using spent and old batteries.^[16]^ In parallel, we evaluated 200 individuals (100 euthyroid aged 47.7 ± 15.0, and 100 hypothyroid aged 50.0 ± 15.5) from the metropolitan area of the Quito District (LE), for whom no report for high lead contamination is reported. The participants were matched according to age and gender. The sample size was defined based on the probability of exposure and OR of 19.27% reported by Pekcici et al^[8]^ obtaining a minimum sample of 43 subjects per group. The demographic and provenance characteristics of participants are presented in Table 1. A venous blood sample of 5 milliliters was collected in free metal tubes, at 8 to 10 am, for the determination of free-thyroxine (FT4), TSH, anti-thyroid peroxidase (anti-TPO), and anti-thyroglobulin (anti-Tg) antibodies. Additionally, a venous blood sample (3 mL) was collected into a tube containing ethylenediaminetetra-acetic acid for lead measurements.

**Table 1:** Demographic features, blood lead levels, and thyroid hormones and thyroid antibodies levels in Ecuadorian euthyroid and hypothyroid individuals, living in high (HE) or low (LE) lead exposure areas.

	All subjects			Euthyroid subjects			Hypothyroid subjects		
		HE area	LE area		HE area	LE area		HE area	LE area
No. of patients, n	384	184	200	197	97	100	187	87	100
Gender, n (%)									
Women	352 (91.7)	174 (94.6)	178 (89.0)	181 (91.9)	92 (94.8)	89 (89.0)	171 (91.4)	82 (94.3)	89 (89.0)
Men	32 (8.3)	10 (5.4)	22 (11.0)	16 (8.1)	5 (5.2)	11 (11.0)	16 (8.6)	5 (5.7)	11 (11.0)
Age, yr (mean ± SD)	50.6 ± 15.0	52.4 ± 14.5	48.9 ± 15.3*	49.6 ± 14.6	51.5 ± 14.1	47.7 ± 15.0	51.6 ± 15.3	53.5 ± 15.1	50.0 ± 15.5
Exposure time to lead, yr (mean ± SD)	37.1 ± 18.3	40.6 ± 19.6	33.9 ± 16.5**	36.8 ± 18.0	39.8 ± 18.7	33.9 ± 16.8#	37.4 ± 18.7	41.4 ± 20.5	33.9 ± 16.2##
Blood lead level, μg/dL, n (%)	5.5 ± 6.7	8.2 ± 8.6	3.0 ± 2.2**	5.8 ± 6.0	8.5 ± 7.4	3.2 ± 2.4**	5.1 ± 7.3	7.8 ± 9.8	2.7 ± 1.9**
≥10 μg/dL	44 (11.5)	42 (22.8)	2 (1.0)	26 (13,2)	25 (25,8)	1 (1.0)	18 (9.6)	17 (19.5)	1 (1.0)
<10 μg/dL	340 (88.5)	142 (77.2)	198 (99.0)	171 (86.8)	72 (74.2)	99 (99.0)	169 (90.4)	70 (80.5)	99 (99.0)
TSH, mUI/L (mean ± SD)	3.6 ± 6.8	4.2 ± 9.6	3.0 ± 2.3	2.8 ± 2.0	2.7 ± 2.2	3.0 ± 1.7	4.4 ± 9.5	5.9 ± 13.5	3.1 ± 2.7
TSH, n (%)									
>4.5 mUI/L	78 (20.3)	36 (19.7)	42 (21.0)	36 (18.3)	15 (15.5)	21 (21.0)	42 (22.5)	21 (24.2)	21 (21.0)
0.45–4.5 mUI/L	281 (73.2)	131 (71.2)	150 (75.0)	155 (78.7)	78 (80.4)	77 (77.0)	126 (67.4)	53 (60.9)	73 (73.0)
<0.45 mUI/L	25 (6.5)	17 (9.1)	8 (4.0)	6 (3.0)	4 (4.1)	2 (2.0)	19 (10.1)	13 (14.9)	6 (6.0)
FT4, ng/dL (mean ± SD)	1.0 ± 0.2	1.0 ± 0.2	1.0 ± 0.2	0.94 ± 0.1	0.93 ± 0.2	0.96 ± 0.1	1.0 ± 0.2	1.0 ± 0.2	1.0 ± 0.2
FT4, n (%)									
<0.9 ng/dL	127 (33.1)	68 (37.0)	59 (29.5)	78 (39.6)	45 (46.4)	33 (33.0)	49 (26.2)	23 (26.4)	26 (26.0)
0.9–1.7 ng/dL	255 (66.4)	114 (62.0)	141 (70.5)	118 (59.9)	51 (52.6)	67 (67.0)	137 (73.3)	63 (72.4)	74 (74.0)
>1.7 ng/dL	2 (0.5)	2 (1.1)	0	1 (0.5)	1 (1.0)	0	1 (0.1)	1 (0.2)	0
Anti-TPO, n (%)									
≥35 U/mL	83 (21.6)	41 (22.3)	42 (21.0)	23 (11.7)	12 (12.4)	11 (11.0)	60 (32.1)	29 (33.3)	31 (31.0)
<35 U/mL	301 (78.4)	143 (77.7)	158 (79.0)	174 (88.3)	85 (87.6)	89 (89.0)	127 (67.9)	58 (66.7)	89 (89.0)
Anti-TG, n (%)									
≥40 U/mL	29 (7.6)	16 (8.7)	13 (6.5)	11 (5.6)	8 (8.2)	3 (3.0)	18 (9.6)	8 (9.2)	10 (10.0)
<40 U/mL	355 (92.4)	168 (91.3)	187 (93.5)	186 (94.4)	89 (91.8)	97 (97.0)	169 (90.4)	79 (90.8)	90 (90.0)

Anti-TG = anti-thyroglobulin antibody, anti-TPO = anti-thyroid peroxidase antibody, FT4 = free-thyroxine, TSH = thyroid stimulating hormone.

* *P* = .02,

** *P* = .001,

# *P* = .021,

## *P* = .007.

Clinical data and blood samples from all individuals were collected after signing an informed consent form, which was approved by the Universidad Central del Ecuador Subcommittee on Ethics in Research in Humans and the Ethics Committee in Research in Human beings at the Eugenio Espejo Specialty Hospital (protocol # MSPCURI0002694).

### 2.2. Lead levels

Pb levels in whole blood were determined at the Department of Clinical, Toxicological, and Bromatological Analysis of the Faculty of Pharmaceutical Sciences of Ribeirão Preto, University of São Paulo. Analyses were performed using inductively coupled plasma mass spectrometry with a quadrupole ion detector (NexION® 2000, PerkinElmer, Waltham, MA).

To verify the accuracy of our data, reference blood samples obtained from the Institut National de Sante` Publique du Quebec in Quebec, Canada were also analyzed as part of the external quality assessment schemes. The obtained values were always in good agreement with the target values (*t* test,95%). The detection limit was 0.02 µg/L. Values <10 μg/dL were considered normal.

### 2.3. Thyroid evaluation

Thyroid function tests were performed at the University Hospital of Ribeirão Preto Medical School, University of São Paulo (USP), Brazil. Measurements of TSH, FT4, anti-TPO, and anti-Tg antibodies were performed using a chemiluminescence method (IMMULITE ® 2000, DPC, Cirrus Inc., Los Angeles, CA) in a single assay. The sensitivity and intra-assay error were 0.02 µU/mL and 3.13% (for TSH), and 0.15 ng/dL and 5.2% for FT4 (analogous method). Reference values within the normal range were defined as: TSH = 0.4 to 4.5 mU/L, FT4 = 0.8 to 1.78 ng/dL, anti-TPO < 35UI/L, and anti-Tg < 40UI/L.

Subclinical hypo/hyperthyroidism was defined as thyroid hormone levels within the normal range and TSH concentrations above or below the defined normal reference range of 0.4 to 4.5 mU/L, respectively.

Thyroid ultrasonography (US) evaluations were performed at the Naval Hospital in Esmeraldas City, PRO-VIDA Hospital of Latacunga City for individuals from Pujilí, and La Merced Clinic in Quito. All US evaluations were performed using a Philips high-resolution ultrasound apparatus (Diamond Select HD11 XE, Bothell, WA). All the patients were classified according to the American College of Radiology - Thyroid Imaging - Reporting and Data System score,^[17]^ by the same radiologist. Fine-needle aspiration biopsy (FNA) was performed in thyroid nodules ≥10 mm with high and intermediate suspicion sonographic pattern according to the 2015 American Thyroid Association guideline^[18]^ and in nodules ≥15 mm and ≥20 mm in those with low suspicion and very low suspicion, respectively. Patients from HE areas were referred to Quito City. FNA was performed by the same radiologist. Cytological results were obtained by a single pathologist and reported using the 2015 BETHESDA system.^[19]^

### 2.4. Statistical analysis

Parametric or non-parametric tests were performed according to the distribution of quantitative variables using the Shapiro–Wilk test, Student T or Mann–Whitney test. Qualitative variables were analyzed using the chi-square test. Inferential statistics were calculated using the Spearman correlation coefficient. Statistical analyses were conducted using the SPSS software (version 22.0; IBM, Chicago, IL), and the level of significance was set at *P < *.05.

The quantitative variables were analyzed using quantitative methods, and grouped according to the reference value as normal, higher and lower than the reference value, for which a qualitative analysis of this grouping was performed.

## 3. Results

186 and 200 subjects were recruited from the high and low exposure areas, respectively. However, 2 HE patients were excluded because they were under 18 years of age. Thus, we applied the data collection and blood sampling form to 384 participants. Due to the pandemic and the mobility of the patients, we performed 300 thyroid ultrasounds. 29 subjects required FNA, however, only in 18 patients were performed.

### 3.1. BPb levels

First, we compared the total BPb levels among all individuals living in areas considered to be highly exposed to lead (Esmeraldas City and the parish of La Victoria) in relation to Quito. The BPb levels observed in the HE areas were higher than those in the LE area (8.2 ± 8.6 vs 3.0 ± 2.26 µg/dL, *P* < .001). In both euthyroid and hypothyroid individuals, the BPb levels were higher in HE when compared to LE areas (8.5 ± 7.4 vs 3.2 ± 2.4 and 7.8 ± 9.8 vs 2.7 ± 1.9, respectively, yielding *P* values < .001 for both comparisons). Comparisons of BPb concentrations in euthyroid and hypothyroid individuals between the HE areas of Esmeraldas and La Victoria revealed no significant differences.

We stratified all studied individuals according to BPb levels, considering high values those >10 μg/dL and low values with BPb <10 μg/dL. In this context, 44 out of 384 individuals (whole group), 26 out of 197 euthyroid individuals, and 18 out of 187 hypothyroid individuals exhibited high BPb levels. Among the individuals exhibiting BPb >10 µg/dL, 42 were from HE and 2 were from LE areas.

HE individuals lived longer in highly exposed areas (39.8 ± 18.7 years) when compared to individuals living in LE areas (33.9 ± 16.8 years, *P* = .02). Hypothyroid individuals living in HE areas lived longer in these areas than euthyroid individuals (*P* = .02). In both hypothyroid and euthyroid individuals, BPb levels correlated positively with lead exposure time; however, this correlation was only significant in hypothyroid patients (*P* = .0003). The major demographic, clinical, and laboratory characteristics of patients are shown in Table 1.

Evaluation of the influence of BPb levels on thyroid function was performed only in euthyroid individuals, as hypothyroid patients were treated with levothyroxine. Euthyroid individuals exhibiting high BPb levels did not present clinical manifestations indicative of thyroid dysfunction. No significant differences were observed in TSH, FT4, and autoantibody levels. Although healthy individuals did not complain and/or present signs of thyroid dysfunction, in the HE area, 15 subjects had TSH values above 4.5 mU/L and 4 subjects had TSH values lower than 0.4 mU/L, values defined as subclinical hypo and hyperthyroidism, respectively. A similar proportion of individuals was observed in the LE area (21 with subclinical hypothyroidism and 2 with subclinical hyperthyroidism) (*P* = .94). Therefore, BPb levels did not influence TSH, FT4, or autoantibody levels in the euthroids (Table 2).

**Table 2:** Thyroid ultrasound and nodule cytological characteristics of Ecuadorian individuals living in high (HE) or low (LE) lead exposure areas.

Variables	All subjects		HE area		LE area		Crude OR (CI 95%)
	n = 300	%	n = 143	%	n = 157	%	
Ultrasound diagnosis							
Nodules	150	50	69	48.2	81	51.6	Reference
Thyroiditis	7	2.3	7	4.9	–	–	0.97 (0.61–1.53)
Normal	143	47.7	67	46.9	76	48.4	
Nodule number							
Solitary	65	42.5	29	40.3	36	44.4	1.19 (0.62–2.26)
Multiple	88	57.5	43	59.7	45	55.6	Reference
Nodule size (n = 309)							
≥1 cm	79	25.6	53	35.1	26	16.5	Reference
<1 cm	230	74.4	98	64.9	132	83.5	2.75 (1.60–4.70)*
ACR-TI-RADS (n = 157)							
TI-RADS 1	40	25.5	23	30.2	17	21.0	1.12 (0.49–2.58)
TI-RADS 2	16	10.2	4	5.3	12	14.8	3.25 (0,82–12.88)
TI-RADS 3	34	21.7	14	18.4	20	24.7	1.55 (0.55–4.38)
TI-RADS 4	42	26.8	22	28.9	20	24.7	0.98 (0.37–2.65)
TI-RADS 5	25	15.9	13	17.1	12	14.8	Reference
Cytological diagnosis (n = 18)							
BETHESDA I	2	11.1	2	16.7	–	–	–
BETHESDA II	14	77.8	10	83.3	4	66.7	–
BETHESDA III	–	–	–	–	–	–	–
BETHESDA IV	1	5.6	–	–	1	16.7	–
BETHESDA V	1	5.6	–	–	1	16.7	–
	*x*	SD	*x*	SD	*x*	SD	*P* value
Thyroid volume	6.5	11.3	11.0	15.6	6.1	3.1	<.001
Nodules per patient	2.0	1.1	2.1	1.1	2.0	1.1	.4
Nodule size	0.7	0.6	1,0	0.8	0.6	0.4	<.001

ACR-TI-RADS = American College of Radiology - Thyroid Imaging - Reporting and Data System, CI = confidence interval, FNA = fine needle aspiration biopsy, OR = odds ratio, SD = standard deviation, x = media.

* *P* < .05.

Thyroid ultrasound was performed in 300 individuals: 143 with HE and 157 with LE. In both euthyroid and hypothyroid individuals: the thyroid volume was greater in the HE zone when compared to LE area (10.2 ± 14.3 vs 6.3 ± 3.1 cm^3^, and 11.8 ± 16.9 vs 2.0 ± 1.1 cm^3^, values *P* < .024 and *P* = .006, respectively), the size of thyroid nodule was increased in individuals from HE areas when compared to LE area (0.9 ± 0.7 cm vs 0.4 ± 0.1, and 0.9 ± 01. vs 0.5 ± 0.1 cm, respectively (*P* < .001 for both comparisons). The number of nodules per individual was only increased in euthyroid subjects from HE area compared to LE areas (2.4 ± 1.2 vs 1.9 ± 1.0, *P* = .04).

Eleven euthyroid subjects (8 from HE and 3 from LE) and 14 hypothyroid patients (5 from HE and 9 from LE) exhibited American College of Radiology - Thyroid Imaging - Reporting and Data System-5 (high suspicion of malignancy). Among the ultrasound-guided FNA procedures performed, only 1 patient from the LE area exhibited TC.

## 4. Discussion

Environmental contaminants have been reported as possible causes of the disruption of thyroid function, influencing the development of benign and malignant thyroid nodules.^[14]^ Several studies have reported an association between lead exposure and thyroid structural and functional changes,^[9]^ as a putative environmental factor. In contrast, others studies have not reported such associations.^[5,13]^ We studied 2 Ecuadorian areas, previously reported to have increased lead exposure, including the sea level city of Esmeraldas and the mountainous parish of La Victoria.^[6]^ Currently, approximately 30,000 inhabitants of the Esmeraldas city have lived in legal and illegal settlements 1 to 5 kilometers from the petrol refinery, being exposed to petroleum derivatives and petroleum waste.^[5]^ Although lead contamination in the parish of La Victoria (around 3000 inhabitants) has been associated with adult workers, laboring on ceramic utensil manufacturing, high levels of lead contaminations have been observed both in infants to adults.^[6]^ In this study, we confirmed that the areas of Esmeraldas and La Victoria did exhibit increased BPb levels when compared to Quito. Indeed, a previous study evaluating 200 children (5–12 years old) performed by our group revealed that more than half of the children living <5 kilometers from the refinery and one-third of the children living <30 km from the refinery exhibited BPb levels of >5 μg/dL (unpublished results). In contrast, increased BPb levels have been reported for more than 30 years in La Victoria,^[6]^ and recent studies have shown that BPb levels decreased from that time to 2015,^[19]^ probably because of population awareness regarding the source of Pb in this area.

Regarding thyroid function, no relationship was found between BPb levels and thyroid function as evaluated using TSH levels. Our results are in agreement with those of previous studies that reported no relationship between lead exposure and TSH concentration in lead smelter workers.^[13,20,21]^ Similarly, this study showed no association between BPb levels and FT4 levels, corroborating the results reported by other groups in children^[22]^ or adults.^[13]^ Finally, a meta-analysis published in 2016 also found no relationship between BPb, T4, and FT4 in lead-exposed and non-exposed groups.^[23]^ In contrast, a study evaluating car battery factory workers reported that TSH and FT4 levels varied according to BPb levels.^[24]^ Qualitative or quantitative analyses of autoantibodies (anti-TPO and anti-Tg) against thyroid antigens revealed no significant differences among the studied populations, yielding similar percentages among individuals living in HE or LE areas. These results were similar to those observed in healthy North American individuals.^[25]^

Few studies have reported the effects of lead on thyroid nodules, particularly in women,^[15]^ and much needs to be learned about the association between nodular goiters and lead contamination. Considering that the majority of the individuals in this study were women, primarily exposed to lead in the house environment (Esmeraldas) and during household manufacturing (Pujilí); individuals from HE areas exhibited larger thyroid volumes and larger thyroid nodules when compared to LE areas in both euthyroid and hypothyroid individuals; hypothyroid individuals lived longer in these areas than euthyroid individuals; and a positive correlation with the duration of environmental exposure was observed only in hypothyroid patients, suggesting a relationship between nodular goiter, hypothyroidism, and Pb exposure. Although the number of thyroid nodules increases with age,^[26]^ further studies are needed to clarify the association between thyroid goiter and Pb contamination.

Despite the large number of thyroid nodules reported, the majority were smaller than 1 centimeter, exhibiting no indication for FNA.^[17]^ Notwithstanding, only 1 TC was observed in a patient from an LE area, covering a non-representative sample for further conclusions regarding the association between lead exposure and TC.

In conclusion, the present study confirmed high exposure to Pb in the locations of Esmeraldas and La Victoria de Pujilí compared with Quito. Although high BPb levels were observed in these areas, no relationship was observed between TSH and FT4 levels and autoantibody levels. Nevertheless, larger thyroid glands, with a greater number of nodules exhibiting larger dimensions, were found in areas with high Pb exposure. Although there has been an improvement in Pb contamination in La Victoria de Pujilí over the years, further measures to control contamination are yet to be implemented. This is the first report to evaluate Pb contamination in Esmeraldas and deserves attention to prevent environmental complications related to Pb exposure.

## Acknowledgments

The authors thank Ms. Rosemeire de Paula Braz, Siemens Healthiness representative, São Paulo, Brazil, for providing kits for measuring TSH, T4L, anti-peroxidase, and anti-thyroglobulin antibodies using the Immulite system. The authors thank Geraldo Cassio Reis, a biostatistician, for analyzing the data and reviewing the manuscript

## Author contributions

**Conceptualization:** José Estefano Rivera-Buse, Léa Maria Zanini Maciel.

**Data curation:** José Estefano Rivera-Buse, Sheila Jissela Patajalo-Villalta, Patrícia Künzle Ribeiro Magalhães.

**Formal analysis:** José Estefano Rivera-Buse, Sheila Jissela Patajalo-Villalta, Léa Maria Zanini Maciel.

**Funding acquisition:** Léa Maria Zanini Maciel.

**Investigation:** José Estefano Rivera-Buse, Sheila Jissela Patajalo-Villalta, Fernando Barbosa Junior, Patrícia Künzle Ribeiro Magalhães.

**Methodology:** José Estefano Rivera-Buse, Léa Maria Zanini Maciel.

**Project administration:** José Estefano Rivera-Buse, Sheila Jissela Patajalo-Villalta.

**Resources:** Léa Maria Zanini Maciel.

**Validation:** Léa Maria Zanini Maciel.

**Visualization:** Patrícia Künzle Ribeiro Magalhães.

**Writing – original draft:** José Estefano Rivera-Buse, Eduardo Antônio Donadi, Léa Maria Zanini Maciel.

**Writing – review & editing:** Eduardo Antônio Donadi, Léa Maria Zanini Maciel.
